# Role of oral-gut microbiota dysbiosis in regulating systemic impairment during age-related obesity: an animal study

**DOI:** 10.3389/fcimb.2026.1781222

**Published:** 2026-02-26

**Authors:** Yixue Tian, Min Yu, Jingxuan Bai, Yuke Chen, Xin Cong, Xuemei Gao

**Affiliations:** 1 Department of Orthodontics, Peking University School and Hospital of Stomatology, Beijing, China; 2 Center for Oral Therapy of Sleep Apnea, Peking University Hospital of Stomatology, Beijing, China; 3 National Center for Stomatology, Beijing, China; 4 Dental Medical Center, China-Japan Friendship Hospital, Beijing, China; 5 Department of Physiology and Pathophysiology, Peking University Health Science Center, Beijing, China

**Keywords:** high-fat diet, obesity, oral microbiota, oral-gut-liver-brain axis, *Romboutsia B*

## Abstract

**Objective:**

To characterize the systemic effects of high-fat diet (HFD)-induced obesity across different ages, explore the microbiota-related obesity endotype using 16S rRNA sequencing, and identify key microbial genera as candidate markers for longitudinal monitoring and future interventional validation.

**Materials and methods:**

Male C57BL/6J mice were randomly assigned to a standard chow diet (SCD) or HFD group, maintained until 4, 12 and 18 months of age as the young, middle-aged and old groups, respectively, at which time animals were euthanized. Systemic effects were evaluated by measuring body weight, Lee’s index, glucose-lipid metabolism, liver function, and blood oxygen levels, coupled with behavioral tests for mood and cognitive performance. Blood samples were collected to quantify LPS and Aβ1–42 levels using ELISA. Oral and fecal samples were collected for 16S rRNA sequencing to analyze microbiota diversity and community structure. Differential genera were identified by LEfSe, and those consistently altered in both oral and gut samples were operationally designated as marker genera. Targeted metabolomics was performed to analyze short-chain fatty acids (SCFAs). Correlations were evaluated using Spearman analysis.

**Results:**

Compared with SCD, HFD mice showed systemic alterations across all age groups, including progressive obesity, elevated blood lipids and liver enzymes, accompanied by reduced blood oxygen, increased Aβ1–42 and LPS levels, increased anxiety-/depression-like behaviors, and impaired spatial memory. HFD significantly remodeled the alpha/beta-diversity and community structure of oral and gut microbiota, inducing stable enrichment of *Romboutsia_B* and depletion of beneficial genera (*Bifidobacterium*, *Akkermansia*, and *Muribaculum*). The abundance of *Romboutsia_B* positively correlated with obesity, blood lipids, liver enzyme levels, hypoxia, and inflammatory markers, but negatively correlated with multiple cognitive-behavioral parameters. Functional prediction and SCFA further profiling indicated that HFD enhanced lipid metabolism and environmental adaptation pathways, while reducing polysaccharide degradation and vitamin metabolism.

**Conclusions:**

Long-term HFD is associated with systemic remodeling of the oral-gut-liver-brain axis across ages. *Romboutsia_B*, a pro-inflammatory–associated genus stably enriched in the oral and gut across all age groups, holds potential as a noninvasive microbial biomarker and candidate target for future intervention studies for obesity and its liver-brain comorbidities.

## Introduction

The prevalence of obesity has increased rapidly worldwide, reaching pandemic levels (Blüher, 2019). Obesity substantially increases the risk of type 2 diabetes, cardiovascular diseases, metabolic syndrome, obstructive sleep apnea, and cancer, thereby increasing the burden on public health systems.

Recently, emerging evidence indicate that the gut microbiota, a major component of the human microbiome, plays pivotal roles in regulating host energy metabolism, internal homeostasis, intestinal barrier integrity, and systemic immune responses, and participates in emotional regulation and neurobehavioral modulation through the gut-brain axis (Cryan and Dinan, 2012; Hsiao et al., 2013; Bárcena et al., 2019). The development of obesity results from a bidirectional interaction between diet and the host’s microbial composition, rather than from merely excess caloric intake (Sasidharan Pillai et al., 2024). Several microbiota transplantation studies have demonstrated that obesity can be induced through gut microbiota (Bäckhed et al., 2004; Turnbaugh et al., 2006; Ridaura et al., 2013), indicating “obese microbiota” possess enhanced capacities for energy extraction and storage (Tilg and Kaser, 2011). Diet can rapidly alter the human gut microbiome (David et al., 2014); therefore, a high-fat diet (HFD) could induce obesity-associated changes in the gut microbiome to control hosts’ metabolism, characterized by shifts in short-chain fatty acid (SCFA) profiles, reduced microbial diversity, and altered metabolic pathways (Turnbaugh et al., 2008; Tilg et al., 2009). On the one hand, a specific microbial profile can increase the efficiency of dietary energy extraction, facilitating the absorption and redistribution of fatty acids and monosaccharides; on the other hand, they modulate metabolic cross-talk among the gut, liver, adipose tissue, and insulin sensitivity, through signaling molecules such as SCFAs, branched-chain amino acids, and aromatic metabolites (DiBaise et al., 2008).

The oral cavity contains the second-largest microbiota in the human body (Rajasekaran et al., 2024; Tian et al., 2024), and its dysbiosis is not only a key driver of periodontal diseases but also contributes to the development of obesity, cardiovascular disorders, gastrointestinal diseases, and Alzheimer’s disease through systemic inflammation, transient bacteremia, and translocation along the oral-gut axis (Peng et al., 2022). Previous studies have found that HFD could induce consistent alterations in the oral and gut microbiome (Bai et al., 2025). However, compared with the relatively well-established research on the gut microbiota, the role of the oral microbiota in obesity and metabolic disorders has long been overlooked, and its synergistic relationship with the gut microbiota remains insufficiently characterized. Additionally, most existing evidence comes from cross-sectional studies, and there is a lack of longitudinal animal research integrating multidimensional phenotypes—including the oral-gut microbiota, metabolism, liver injury, hypoxia, and neurobehavior—within a unified experimental framework.

Therefore, in this study, we established HFD-induced obesity mouse models across multiple age groups, and employed 16S rRNA sequencing technology to (1) characterize the systemic consequences of obesity with aging; (2) investigate the microbiota-driven obesity endotypes associated with increasing age; and (3) identify marker genera that may serve as candidate biomarkers and hypothesis-generating taxa for future mechanistic/interventional studies.

## Materials and methods

### Study design

Five-week-old male C57BL/6J mice were used in this study, which were housed in a specific pathogen-free (SPF)-grade barrier facility. Throughout the experiment, the animals had ad libitum access to irradiated chow and autoclaved drinking water. All procedures involving animals were conducted in accordance with the guidelines of the Peking University Animal Research Committee and were approved by the Animal Protection and Use Committee of Peking University (Approval No. DLASBD0276).

**Figure 1:**
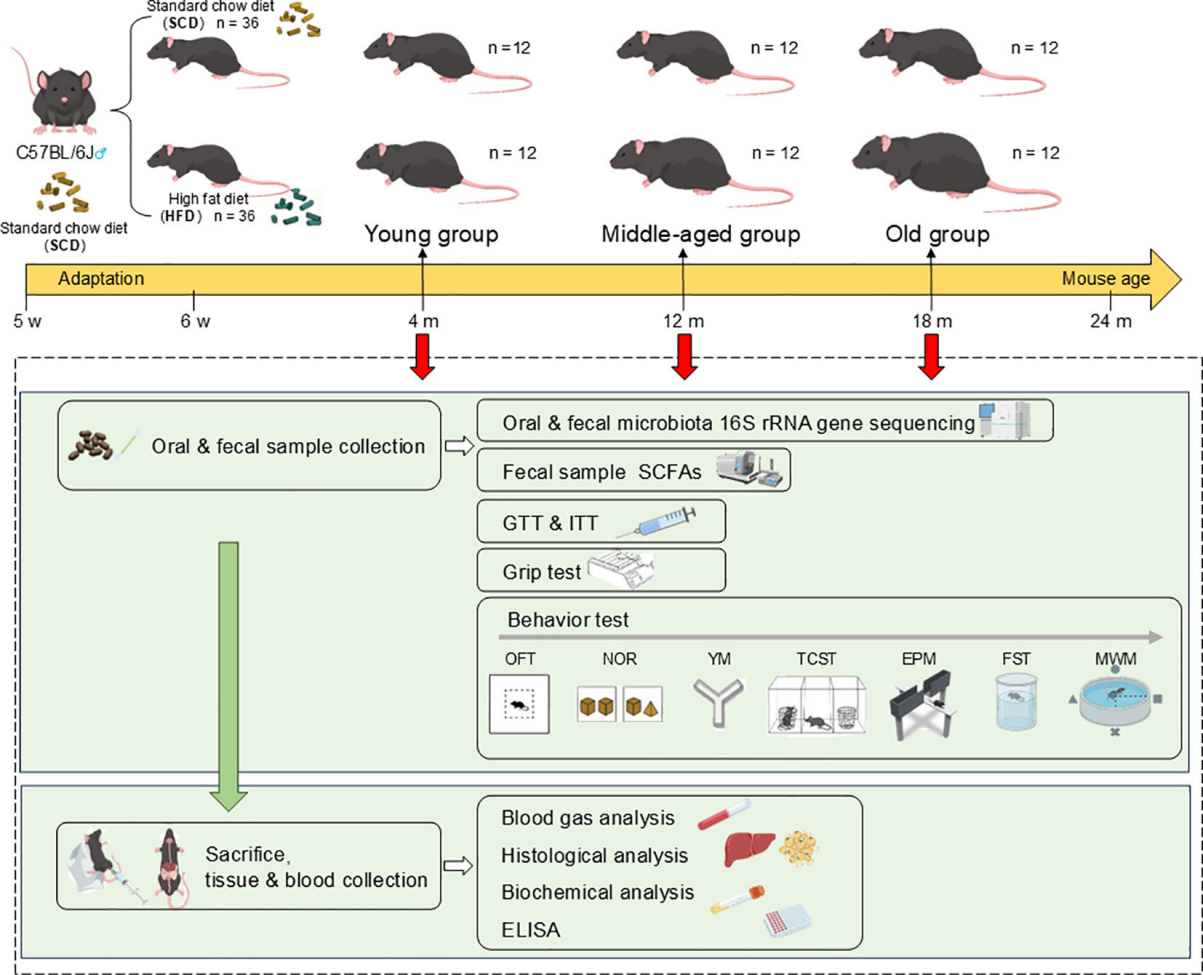
Overview of the study design. GTT, Glucose tolerance test; ITT, insulin tolerance test; OFT, open field test; NOR, novel object recognition test; YM, Y-maze; TCST, three-chamber social test; EPM, elevated plus maze; FST, forced swim test; MWM, Morris water maze. Created with (Jiang et al., 2024).

The study design is illustrated in **Figure 1**. After one week of acclimatization, the mice were randomly divided into either the standard chow diet (SCD) group or the high-fat diet (HFD) group, with body weight recorded weekly. The HFD used was Research Diets D12492 (60% kcal fat, 20% kcal protein, 20% kcal carbohydrate), and detailed formulations and sourcing information for both the SCD and HFD are provided in the **Supplementary File 1**. Previous studies have indicated that mice with a body weight exceeding 120% of that of age-matched controls can be defined as obese (Wen et al., 2024; Zhang et al., 2024). Based on the survival curve of C57BL/6J mice and the mouse-human age equivalence chart (Flurkey et al., 2007), mice aged 3 to 6 months, 10 to 14 months, and 18 to 24 months were categorized as mature adult, middle-aged, and old, respectively, roughly corresponding to 20 to 30 years, 38 to 47 years, and 56 to 69 years of age in humans. To investigate the systemic effects of a HFD on the oral and gut microbiota, glucose and lipid metabolic capacity, and emotional and cognitive functions, mice were randomly selected from the HFD and SCD groups at 4, 12, and 18 months of age (defined as the young, middle-aged, and old groups, respectively). After an overnight fast, oral and fecal samples were collected, followed by glucose tolerance and insulin sensitivity tests. Subsequently, forelimb grip strength testing and a series of behavioral assessments were then conducted. Finally, the mice were subsequently euthanized at the corresponding timepoints (4, 12, and 18 months of age), and liver, adipose tissue, and blood samples were collected.

### Oral and fecal sample collection and microbiota analysis

Mice were fasted overnight for 14 to 16 hours before oral and fecal sample collection, and all samples were uniformly collected between 9:00 and 11:00 AM the next morning. Following previous studies (Abusleme et al., 2017, Abusleme et al., 2020; Bai et al., 2025), sterile cotton swabs were used to gently swab the oral mucosa of the mice, and each swab was immediately placed into a 1.5 mL sterile microcentrifuge tube containing 200 μL of Tris-EDTA buffer. Approximately 20 mg of freshly excreted feces was collected into a 1.5 mL sterile tube. Both oral and fecal samples were transported on ice and stored at -80 °C within 2 hours to preserve microbial integrity.

Genomic DNA of the oral microbiota was extracted using the Cetyltrimethylammonium Bromide (CTAB) method (Dong et al., 2019), while nucleic acids from the gut microbiota were extracted using the MagBeads FastDNA Kit for Soil. The extracted DNA was assessed by 0.8% agarose gel electrophoresis to evaluate molecular size, and quantified using a Nanodrop micro-spectrophotometer. PCR amplification targeted the bacterial 16S rRNA V3-V4 region using the specific primers 338F (5′-ACTCCTACGGGAGGCAGCA-3′) and 806R (5′-GGACTACHVGGGTWTCTAAT-3′). PCR products were quantified and pooled using the Quant-iT PicoGreen dsDNA Assay Kit. Library preparation was performed using the TruSeq Nano DNA LT Library Prep Kit (Illumina). Libraries were quantified fluorometrically and quality-controlled with a Bioanalyzer to verify the size distribution of PCR-enriched fragments. DNA libraries were normalized to 10 nmol/L, pooled in equal volumes, and subjected to paired-end 2×250 bp sequencing on an Illumina NovaSeq 6000 platform. Raw sequencing data were stored in FASTQ format.

Microbiome bioinformatic analyses were performed using QIIME2. Sequences were demultiplexed and processed using the DADA2 workflow (Callahan et al., 2016) for quality filtering, denoising, merging, and chimera removal, generating amplicon sequence variants (ASVs) and abundance tables. Taxonomic annotation was performed using the Greengenes2 database (McDonald et al., 2024).

### Targeted metabolomics analysis by gas chromatography-mass spectrometry

Twenty mg of fecal sample was weighed into a centrifuge tube after thawing, mixed with stainless steel beads and 1,000 μL of 0.5% (v/v) phosphate solution. The mixture was homogenized and then centrifuged. The supernatant was then extracted with 500 μL of methyl tert-butyl ether (MTBE; CNW Technologies) containing internal standards, vortexing, ultrasonication, and centrifugation, the supernatant was then collected (Bianchi et al., 2011; Lotti et al., 2017; Primec et al., 2017; Kim et al., 2022). Targeted quantification of fecal short-chain fatty acid (SCFAs) was performed using a gas chromatography-mass spectrometry system (Agilent 8890-7000D). The sum of acetate, propionate, butyrate, valerate, and caproate abundance was defined as total straight-chain SCFAs (scSCFAs), while the sum of isobutyrate, isovalerate, and 2-methylbutyrate abundance was defined as total branched-chain SCFAs (bcSCFAs). After internal standard correction, SCFA abundance was normalized to sample weight and expressed as mg/g.

### Glucose tolerance test and insulin tolerance test

After overnight fasting with free access to water for 14 to 16 hours, blood was collected from the tail vein, and fasting blood glucose was measured using a glucometer (Sinocare GA-3). Mice were then intraperitoneally injected with 25% glucose solution (2 g/kg), and blood glucose levels were measured at 15, 30, 60, 90, and 120 minutes post-injection.

One week after the GTT, mice were fasted with free access to water for 4 to 6 hours. Fasting blood glucose was measured via tail-vein sampling, followed by an intraperitoneal injection of insulin (0.75 U/kg). Blood glucose levels were recorded at 15, 30, 60, 90, and 120 minutes after insulin injection.

### Grip strength test

Forelimb grip strength was assessed using a grip strength meter (YLS-13A). After each trial, mice were allowed to rest for 1 minute. Each mouse was tested three times, and the average value was used as its final grip strength.

### Behavior tests

A series of behavioral tests were conducted to evaluate locomotor activity, anxiety-like behavior, cognitive function, social interaction, and depression-like behavior in mice (Vorhees and Williams, 2006; Kaidanovich-Beilin et al., 2011; Can et al., 2012; Lu et al., 2018; Kraeuter et al., 2019; Smith et al., 2022; Zhuang et al., 2022; Zhao et al., 2023). Behavioral testing and scoring were conducted by investigators blinded to group allocation, with a fixed test order and predefined inter-assay intervals (24 or 48 h); detailed procedures are provided in **Supplementary File 2**. Briefly, anxiety-like behavior was initially assessed using the Open Field Test (OFT) by quantifying the distance traveled in the center zone, time spent in the center, and number of entries into the center. Short-term recognition memory was evaluated via the Novel Object Recognition (NOR) test, and the discrimination index (DI) was calculated. Working memory was measured in the Y-maze based on the percentage of spontaneous alternations. Social behavior and social novelty preference were examined using the Three-Chamber Social Test (TCST), generating both sociability and social novelty indices. Anxiety-like behavior was further assessed using the Elevated Plus Maze (EPM) by recording open- and closed-arm entries and exploration time. Depression-like behavior was evaluated using the Forced Swim Test (FST) by quantifying immobility time. Spatial learning and hippocampus-dependent memory were tested in the Morris Water Maze (MWM), including acquisition latency to the hidden platform, probe trial swim trajectories, platform crossings, and time spent in the target quadrant.

### Oxygen saturation measurement and blood gas analysis

Mice were anesthetized with 1.5% isoflurane (Liu et al., 2024), and a pulse oximeter probe (M8001A, Philips) was clipped onto the shaved left thigh. After the readings stabilized, peripheral oxygen saturation (SpO_2_) was recorded.

For arterial blood gas analysis, mice were anesthetized with 3% isoflurane for 3 minutes. After opening the abdominal cavity, 100 μL of arterial blood was collected from the abdominal aorta using a heparinized 1 mL syringe. The sample was immediately loaded into a blood gas analyzer (ABL80 FLEX, Radiometer) for measurement (Song et al., 2019; Wang et al., 2024).

### Tissue and blood sample collection and analysis

After completion of all behavioral tests, mice were fasted overnight and deeply anesthetized the next morning at 9:00 AM with sodium pentobarbital (80 mg/kg, i.p.) for terminal cardiac blood collection, followed by cervical dislocation to ensure euthanasia. Mice were then dissected, and visceral adipose tissue, liver, and brain tissues were harvested. Liver and adipose tissues were fixed in 4% paraformaldehyde for 48 hours. Brain tissues were rapidly frozen in liquid nitrogen and stored together with serum samples at -80 °C for further analyses.

Liver and adipose tissues were subjected to hematoxylin-eosin (HE) staining, and morphological alterations were examined under a light microscope (E100/CX23, Nikon/Olympus). Liver tissue was also processed for Oil Red O staining and quantitative analysis.

A fully automated biochemical analyzer (BS-350E) was used to measure serum levels of total cholesterol (TC), high-density lipoprotein cholesterol (HDL-C), low-density lipoprotein cholesterol (LDL-C), triglycerides (TG), glucose (Glu), alanine aminotransferase (ALT), and lactate dehydrogenase (LDH).

### ELISA

Serum concentrations of amyloid β 1-42 (Aβ1-42), S100 calcium-binding protein β (S100β), tumor necrosis factor-α (TNF-α), interleukin-6 (IL-6), and IL-10 were quantified using mouse ELISA kits. Additionally, levels of Aβ1-42, TNF-α, IL-6, IL-10, and lipopolysaccharide (LPS) in the cerebral cortex, as well as Aβ1–42 in the hippocampus, were also measured. Mouse ELISA kits from Elabscience and MultiSciences were used for these detections.

### Bioinformatics and statistical analysis

Alpha-diversity metrics, including Chao1 and Good’s coverage, were calculated to evaluate within-sample richness and evenness. Between-sample differences (beta-diversity) were assessed using principal coordinate analysis (PCoA) based on the Jaccard distance. Hierarchical clustering was conducted using the unweighted pair-group method with arithmetic means (UPGMA) based on the same distance matrix. Group-level differences and within-group dispersion were evaluated using permutational multivariate analysis of variance (PERMANOVA) and permutational analysis of multivariate dispersions (PERMDISP), respectively, with 999 permutations each. All plots were generated in R (v4.3.0, Austria). Differentially enriched taxa with biological relevance were identified through linear discriminant analysis effect size (LEfSe). Functional potential of gut microbial communities was inferred using PICRUSt2 (v2.2.0), and predicted pathways were annotated using the Kyoto Encyclopedia of Genes and Genomes (KEGG) database.

All variables were continuous. Normality was assessed using the Shapiro-Wilk test. Normally distributed data are presented as mean ± SD and analyzed with parametric tests; non-normal data are reported as median (interquartile range, IQR) and analyzed using nonparametric tests. All tests were two-sided with significance set at *P* < 0.05. For single-factor comparisons, an independent-samples t-test or one-way analysis of variance (ANOVA) with Tukey’s honestly significant difference (HSD) *post hoc* test was used when assumptions were met; Welch’s t-test or Welch ANOVA with Dunnett T3 *post hoc* test was applied for unequal variances; Mann-Whitney U and Kruskal-Wallis H tests with Dunn *post hoc* test and false discovery rate (FDR) correction were used for non-normal data. For two-factor analyses, two-way ANOVA with Tukey HSD or Dunnett *post hoc* test was used when assumptions were met. All statistical analyses were performed using GraphPad Prism (v10.1.2) and SPSS (v27.0, IBM).

## Results

### Effects of high-fat diet and aging on physiological parameters

**Figure 2:**
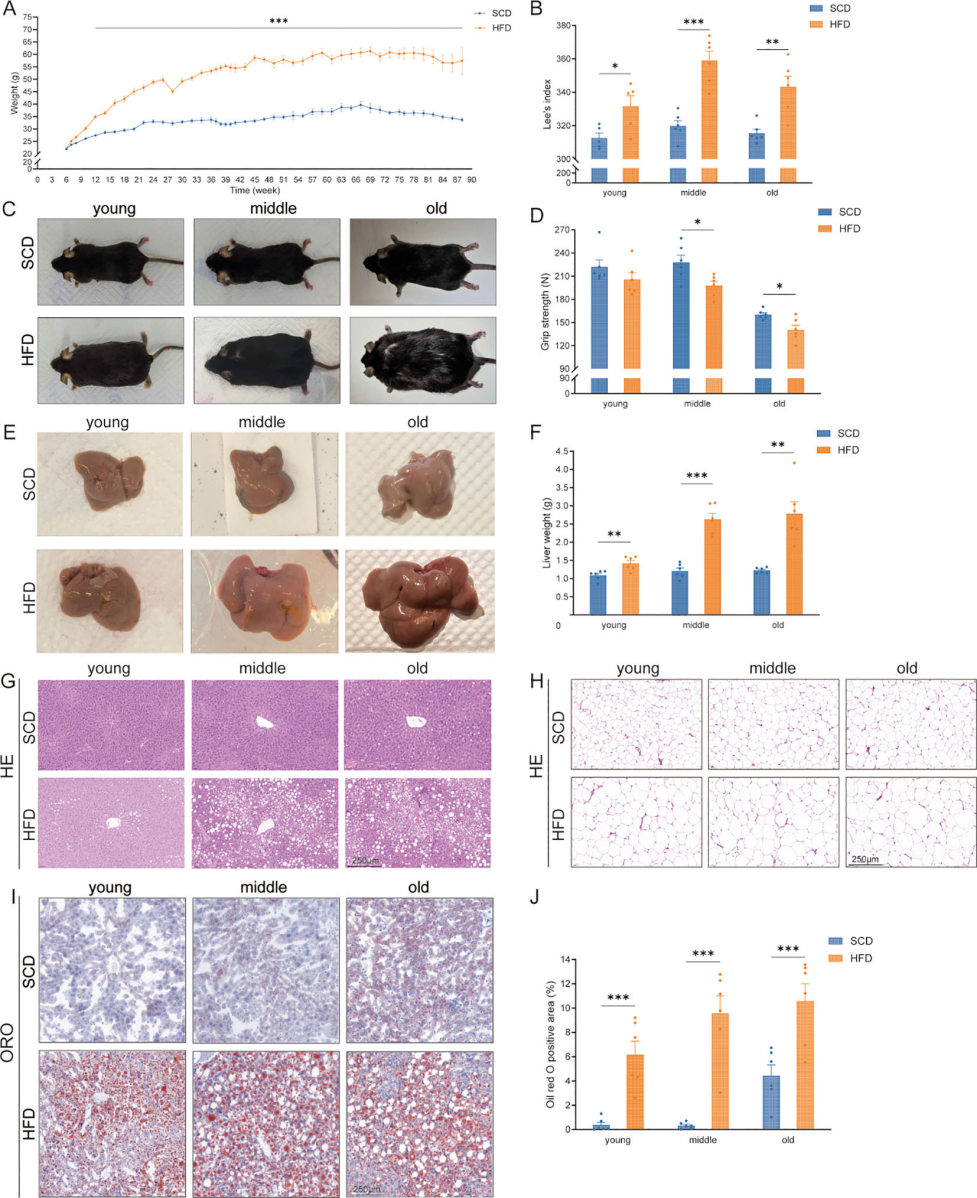
Effects of a high-fat diet and aging on weight, liver, and adipose tissue. **(A)** Body weight changes in mice under HFD and SCD; **(B)** Comparison of Lee’s index among mice in the three age groups; **(C)** Representative dorsal gross images of mice in the young, middle-aged, and old age groups; **(D)** Comparison of forelimb grip test in the three age groups; **(E)** Representative gross images of livers in the young, middle-aged, and old age groups; **(F)** Comparison of liver weight in the three age groups. **(G)** HE staining of liver tissue in the young, middle-aged, and old age groups (scale bar = 250 μm); **(H)** HE staining of adipose tissue from mice in the young, middle-aged, and old age groups (scale bar = 250 μm); **(I)** Oil Red O staining of liver tissue in the young, middle-aged, and old age groups (scale bar = 250 μm); **(J)** Quantification of the percentage of Oil Red O-positive area in liver sections in the three age groups. HFD: high-fat diet; SCD: standard chow diet. *P < 0.05, **P < 0.01, ***P < 0.001.

During the experiment, body weight gain in the HFD group was consistently higher than in the SCD group. Two-way ANOVA showed significant main effects of diet [F(1, 929) = 4569, *P* < 0.0001] and age [F(45, 929) = 65.9, *P* < 0.0001], as well as a significant interaction [F(45, 929) = 14.54, *P* < 0.0001]. *Post hoc* comparisons revealed that from week 12 onward, HFD mice had significantly higher body weight than SCD controls (**Figure 2A**, *P* < 0.001). Body length measurements and Lee’s index were significantly higher in HFD mice (**Figure 2B**, *P* < 0.05). Representative dorsal images of mice in the young, middle-aged, and old groups are shown in **Figure 2C**. Forelimb grip strength did not differ between HFD and SCD mice in the young group, whereas HFD mice in the middle-aged and old groups displayed significantly reduced grip strength (**Figure 2D**, *P* < 0.05).

**Figure 2E** shows representative images of livers from mice in the young, middle-aged, and old age groups. Liver weights were significantly higher in the HFD group compared with the SCD group across all age groups (**Figure 2F**, *P* < 0.01). HE staining of liver tissue (**Figure 2G**) revealed that, relative to age-matched SCD controls, livers from HFD mice exhibited disorganized hepatic cords, hepatocyte enlargement, large cytoplasmic vacuoles displacing nuclei to the cell periphery, and inflammatory foci; scattered hepatocyte necrosis was observed in the old HFD group. HE staining of adipose tissue (**Figure 2H**) demonstrated that, compared with age-matched SCD mice, HFD mice had larger adipocytes, more scattered inflammatory cells, and higher cellular heterogeneity. In the old HFD group, mixed clusters of adipocytes of varying sizes were observed, with progressive thickening of the interstitial tissue and formation of crown-like structures. Oil Red O staining of liver tissue showed a marked increase in positively stained area in HFD mice compared with age-matched SCD controls (**Figures 2I, J**, *P* < 0.001).

**Figure 3:**
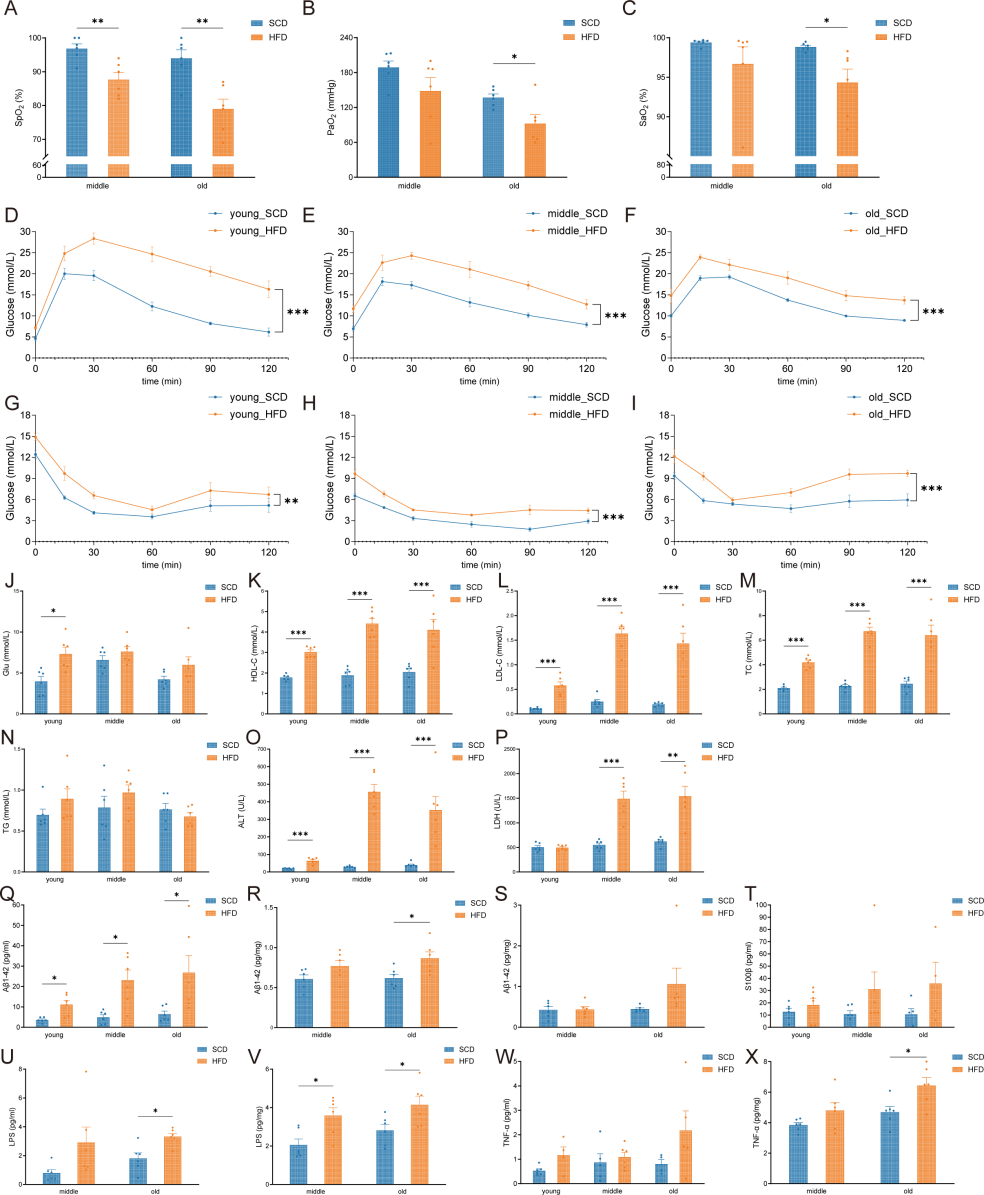
Effects of a high-fat diet and aging on systemic effects. **(A-C)** Comparison of pulse oxygen saturation [SpO_2_; **(A)**], arterial oxygen partial pressure [PaO_2_; **(B)**], and arterial oxygen saturation [SaO_2_; **(C)**] in mice; **(D-F)** Glucose tolerance tests (GTT) in mice from the young, middle-aged, and old age groups; **(G-I)** Insulin sensitivity tests (ITT) from the young, middle-aged, and old age groups; **(J-P)** Indices of glucose and lipid metabolism and liver enzymes in mice: fasting blood glucose **(J)**, serum HDL-C **(K)**, LDL-C **(L)**, total cholesterol [TC; **(M)**], triglycerides [TG; **(N)**], alanine aminotransferase [ALT; **(O)**], and lactate dehydrogenase [LDH; **(P)**]; **(Q–S)** Levels of Aβ1–42 in serum **(Q)**, hippocampus **(R)**, and cortex **(S)**; **(T)** serum S100β; **(U, V)** LPS levels in serum **(U)** and cortex **(V)**; **(W, X)** TNF-α levels in serum **(W)** and cortex **(X)**. *P < 0.05, **P < 0.01, ***P < 0.001.

Blood oxygen measurement showed that SpO_2_ was lower in HFD mice than in SCD controls in middle-aged and old age groups (**Figure 3A**, *P* < 0.01). In the old group, arterial oxygen partial pressure (PaO_2_) and oxygen saturation (SaO_2_) were significantly reduced in HFD mice compared with SCD mice (**Figures 3B, C**, *P* < 0.05), whereas no significant differences in PaO_2_ or SaO_2_ were observed between HFD and SCD mice in the middle-aged group. Detailed blood gas parameters are presented in **Supplementary File 3**.

As for glucose and lipid metabolism, HFD mice exhibited significantly elevated GTT curves compared with SCD controls in all three age groups (**Figures 3D-F**, *P* < 0.001), with higher peak values and slower return to baseline, indicating impaired glucose tolerance in HFD mice. Following insulin injection, blood glucose levels in HFD mice decreased significantly less than in SCD mice across all age groups (**Figures 3G-I**, *P* < 0.01), suggesting reduced insulin sensitivity induced by HFD. Serum glucose in the young HFD group was significantly higher than in the young SCD group (*P* < 0.05), whereas no significant inter-group differences were observed in the middle-aged and old groups (**Figure 3J**).

Serum HDL-C, LDL-C, and TC were significantly higher in HFD mice than in SCD controls across all age groups (**Figures 3K-M**, *P* < 0.001), while TG levels did not differ significantly (**Figure 3N**). Serum ALT levels were significantly elevated in HFD mice in all age groups (**Figure 3O**, *P* < 0.001). Serum LDH did not differ between young HFD and SCD mice, but was significantly higher in HFD mice in the middle-aged and old groups (**Figure 3P**, *P* < 0.01).

Regarding neuroinflammation and neural injury markers, HFD mice exhibited significantly higher serum Aβ1–42 across all age groups compared with SCD controls (**Figure 3Q**, *P* < 0.05). Cortical Aβ1–42 was elevated in the old HFD group (**Figure 3R**, *P* < 0.05), while hippocampal Aβ1–42 showed no significant differences (**Figure 3S**). S100β levels were similar between HFD and SCD in all age groups (**Figure 3T**). Serum LPS was increased in the old HFD group (**Figure 3U**, *P* < 0.05), and cortical LPS was higher in middle-aged and old HFD mice (**Figure 3V**, *P* < 0.05). Serum TNF-α did not differ between groups, but cortical TNF-α was elevated in the old HFD group (**Figures 3W, X**, *P* < 0.05). IL-6 and IL-10 levels in serum and cortex were non-significant (**Supplementary File 4**).

### Effects of high-fat diet and aging on emotion and cognitive memory

Two-way ANOVA across all behavioral tests revealed that diet significantly influenced anxiety-like, social, and cognitive behaviors, while age and diet × age interaction affected specific measures, including spontaneous alternation, immobility, and spatial memory.

**Figure 4:**
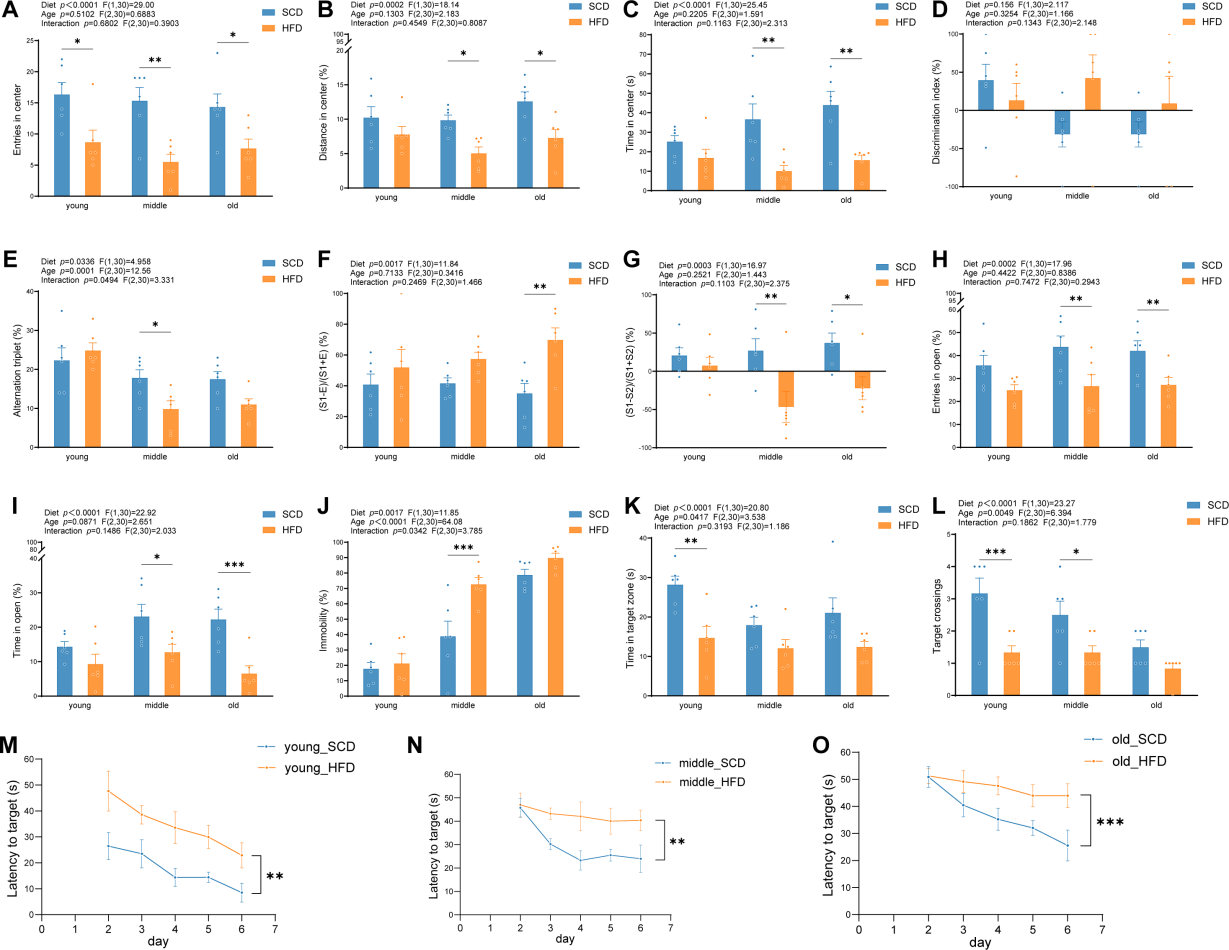
Effects of high-fat diet and aging on emotion and cognitive memory. **(A-C)** Open field test (OFT): **(A)** Number of entries into the central area of the open field; **(B)** Percentage of distance traveled in the central area relative to the total distance; **(C)** Percentage of time spent in the central area relative to the total test duration; **(D)** Novel object recognition (NOR) test: discrimination index; **(E)** Y-maze (YM) test: percentage of spontaneous alternations; **(F, G)** Three-chamber social test (TCST); **(F)** Social preference index for the social target versus the empty target; **(G)** Social novelty preference index for the familiar versus the novel conspecific; **(H, I)** Elevated plus maze (EPM); **(H)** Percentage of entries into the open arms; **(I)** Percentage of time spent in the open arms; **(J)** Forced swim test (FST): percentage of immobility time; **(K-O)** Morris water maze (MWM); **(K)** Time spent swimming in the target quadrant during the probe test; **(L)** Number of platform crossings during the probe test; **(M-O)** Escape latency during the training phase in mice from the young, middle-aged, and old age groups. *P < 0.05, **P < 0.01, ***P < 0.001.

In the OFT, HFD mice made fewer central entries, and middle-aged and old HFD mice traveled shorter distances and spent less time in the center than SCD controls (**Figures 4A-C**, *P* < 0.05), indicating increased anxiety. In the NOR test, the discrimination index was not affected by diet, age, or their interaction (**Figure 4D**). In the Y-maze, middle-aged HFD mice showed reduced spontaneous alternations compared with SCD mice (**Figure 4E**, *P* < 0.05), reflecting impaired short-term spatial working memory. In the TCST, old HFD mice preferred social targets more, and middle-aged and old HFD mice showed a higher preference for familiar conspecifics than SCD mice (**Figures 4F, G**, *P* < 0.05), suggesting a shift in social motivation toward maintaining established relationships. In the EPM, middle-aged and old HFD mice entered open arms less frequently and spent less time in them than SCD mice (**Figures 4H, I**, *P* < 0.05), reflecting increased anxiety. In the FST, middle-aged HFD mice spent more time immobile (**Figure 4J**, *P* < 0.001), indicating enhanced depression-like behavior. In the MWM, young HFD mice spent less time in the target quadrant and had fewer platform crossings (**Figures 4K, L**, *P* < 0.05), whereas these differences were not significant in old mice, suggesting that aging may mask HFD-induced spatial learning deficits. During training, HFD mice also showed longer escape latencies (**Figures 4M-O**, *P* < 0.01).

### Effects of high-fat diet and aging on oral and gut microbiota

**Figure 5:**
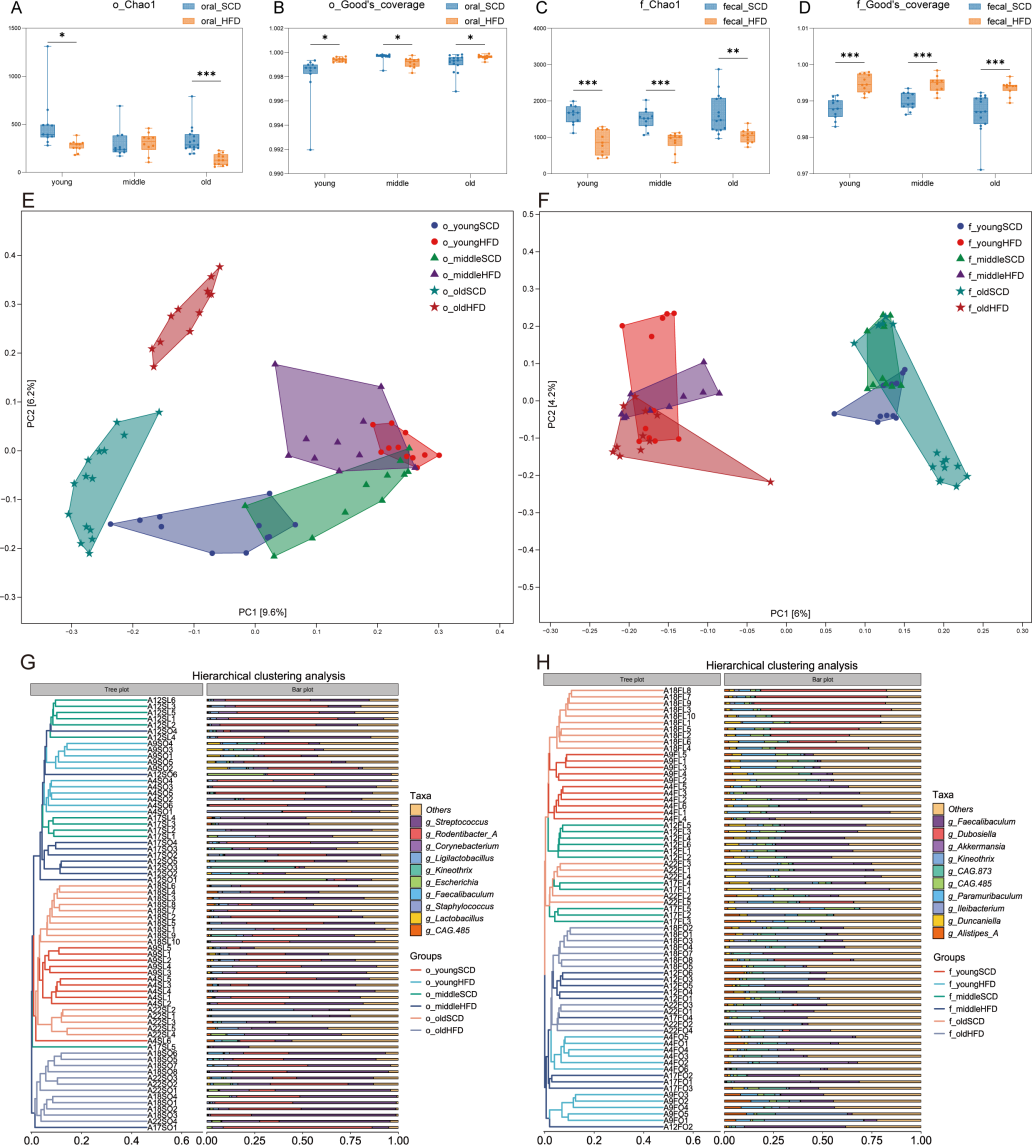
Effects of high-fat diet and aging on oral and gut microbiota. **(A, B)** Alpha-diversity indices of the oral microbiota in mice, represented by Chao1 and Good’s coverage; **(C, D)** Alpha-diversity indices of the gut microbiota in mice, represented by Chao1 and Good’s coverage; **(E)** Principal coordinates analysis (PCoA) plot of the mouse oral microbiota based on Jaccard distance; **(F)** Principal coordinates analysis (PCoA) plot of the mouse gut microbiota based on Jaccard distance; **(G)** UPGMA hierarchical clustering dendrogram of the mouse oral microbiota based on Jaccard distance; **(H)** UPGMA hierarchical clustering dendrogram of the mouse gut microbiota based on Jaccard distance. *P < 0.05, **P < 0.01, ***P < 0.001.

Alpha-diversity of oral and gut microbiota in HFD and SCD mice is shown in **Figures 5A-D**. Compared with age-matched SCD controls, the oral microbiota Chao1 index was significantly reduced in young and old HFD mice (**Figure 5A**, *P* < 0.05), while Good’s coverage index was significantly increased in all three HFD age groups (**Figure 5B**, *P* < 0.05). For gut microbiota, the Chao1 index was significantly lower and Good’s coverage index significantly higher in HFD mice across all age groups compared with SCD mice (**Figures 5C, D**, *P* < 0.01).

PCoA based on Jaccard distance was performed (**Figures 5E, F**), and differences in microbial community structure were assessed using PERMANOVA for between-group variation and PERMDISP for within-group dispersion. The results showed significant differences in beta-diversity between HFD and SCD mice in oral microbiota across all age groups (*P* = 0.001), with no significant within-group differences (*P* = 0.114). Similarly, gut microbiota displayed significant between-group differences in beta-diversity (*P* = 0.001), while within-group differences remained non-significant (*P* = 0.225). The UPGMA demonstrated clear clustering tendencies within each group for both oral and gut microbial communities (**Figures 5G, H**). These findings indicate that a high-fat diet induces marked and widespread alterations in microbial community composition.

PICRUSt2-based KEGG functional annotation (**Supplementary File 5**) revealed that the microbiota in the HFD group was significantly enriched in energy- and lipid metabolism-related pathways, including butyrate metabolism, fatty acid biosynthesis, unsaturated fatty acid metabolism, and ketone body metabolism, whereas the SCD group showed greater enrichment in pathways related to nutrient metabolism and community homeostasis, such as polysaccharide degradation, pentose and glucuronate interconversions, and vitamin metabolism. In parallel, the HFD group also exhibited enrichment of pathways involved in DNA repair, bacterial chemotaxis, and multiple antibiotic resistance and infection-related processes, suggesting that a high-fat diet drives functional remodeling of the microbiota toward enhanced environmental stress adaptation, motility, and pathogenic potential.

### Marker genera in oral and gut microbiota

**Figure 6:**
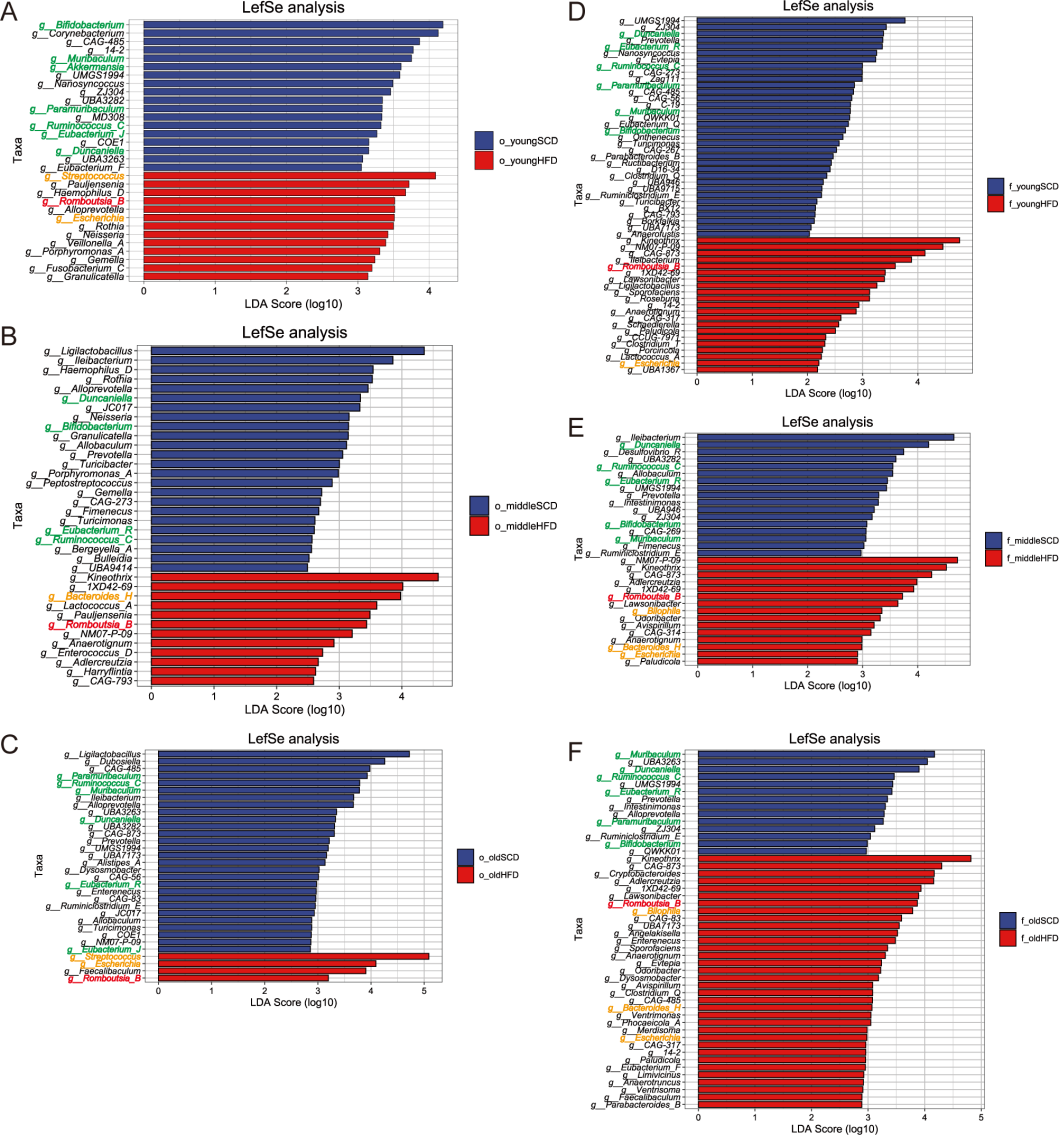
Marker genera in oral and gut microbiota by LEfSe analysis. **(A-C)** Significantly enriched taxonomic genera in the oral microbiota of mice in the young, middle-aged, and old groups; **(D-F)** Significantly enriched taxonomic genera in the gut microbiota of mice in the young, middle-aged, and old groups. *Romboutsia_B* in red; pro-inflammatory–associated genera significantly enriched in the HFD group in yellow; and protective-associated genera significantly enriched in the SCD group in green.

**Figure 7:**
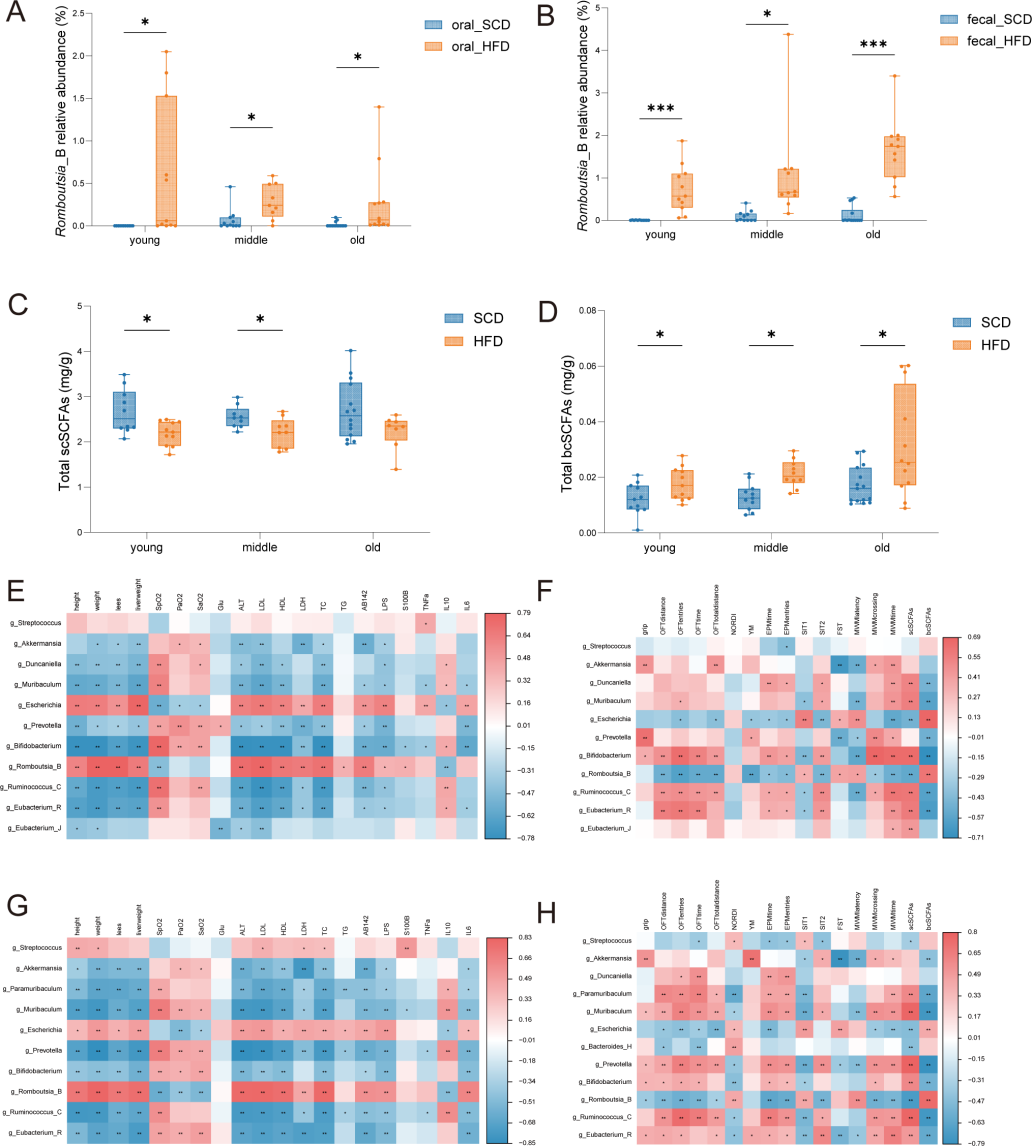
Correlation analysis of marker genera, physiological parameters, SCFAs and behavioral tests **(A, B)** Comparison of the relative abundance of *Romboutsia_B* in the oral and gut microbiota; **(C)** Abundance of total straight-chain short-chain fatty acids (scSCFAs) in feces; **(D)** Abundance of total branched-chain short-chain fatty acids (bcSCFAs) in feces. **(E)** Correlations between differential oral microbial genera and mouse physiological parameters; **(F)** Correlations between differential oral microbial genera with behavioral test performance and fecal SCFA abundances; **(G)** Correlations between differential gut microbial genera and mouse physiological parameters; **(H)** Correlations between differential gut microbial genera with behavioral test performance and fecal SCFA abundances; *P < 0.05, **P < 0.01, ***P < 0.001.

Using an LDA score > 2 and *P* < 0.05, LEfSe analysis identified distinct microbial taxa between HFD and SCD mice on the genus level (**Figures 6A–F**). Across all age groups and in both oral and gut samples, *Romboutsia_B* was consistently enriched in HFD mice (**Figures 7A, B**), whereas *Ruminococcus_C*, *Duncaniella*, and *Eubacterium* were enriched in SCD mice. Several putative pathobiont- and inflammation-associated genera (*Escherichia*, *Streptococcus*, *Bilophila*, *Bacteroides_H*) repeatedly appeared in HFD mice, whereas health- and homeostasis-associated genera (*Bifidobacterium*, *Akkermansia*, *Muribaculum*, *Paramuribaculum*) were enriched in SCD mice.

### Effects of high-fat diet and aging on short-chain fatty acids

The profiles of straight-chain and branched-chain SCFAs in feces exhibited opposite changes under HFD conditions. In the young and middle-aged groups, scSCFAs were significantly lower in the HFD group than in the SCD group (*P* < 0.05), whereas no significant difference was observed between the two groups in the old group (**Figure 7C**). In contrast, bcSCFAs were significantly increased in the HFD group across all three age groups (**Figure 7D**, *P* < 0.05). Taken together, functional prediction and SCFA profiling support an HFD-associated reprogramming of colonic microbial fermentation, characterized by increased production of bcSCFAs and reduced polysaccharide degradation capacity.

Spearman correlation analysis was performed to explore associations between differentially abundant taxa and various physiological and behavioral tests. *Romboutsia_B* and *Escherichia* showed positive correlations with body length, body weight, Lee’s index, ALT, LDH, HDL-C, LDL-C, TC, Aβ1-42, S100β, LPS, TNF-α, IL-6, and bcSCFAs. These genera were also positively correlated with social preference index, immobility time in the FST, and escape latency in the Morris water maze. In contrast, they were negatively correlated with SpO_2_, PaO_2_, SaO_2_, IL-10, and scSCFAs, as well as forelimb grip strength, time and distance spent in the center zone in the OFT, spontaneous alternation in the Y-maze, time and entries into the open arms in the EPM, social novelty preference, and platform crossings and probe-trial performance in the Morris water maze (**Figures 7E-H**). Conversely, *Ruminococcus_C*, *Duncaniella*, *Eubacterium*, *Bifidobacterium*, *Akkermansia*, *Muribaculum*, and *Prevotella* displayed opposite correlation patterns. These results were highly consistent between oral and gut microbiota.

## Discussion

This study established a long-term HFD-induced obesity model spanning early adulthood to old age, covering the full adult lifespan of mice. We evaluated the systemic impacts of obesity and aging, including hepatic and adipose histopathology, blood gas parameters, inflammation, Aβ burden, and multidimensional behavioral phenotypes, while simultaneously profiling oral and gut microbiota using 16S rRNA sequencing and bioinformatic analyses. Targeted fecal SCFA metabolomics were integrated to provide a multi-omics perspective. Age was considered an effect-modifying biological context, and HFD–SCD contrasts were interpreted within and across young, middle-aged, and old cohorts to capture life-stage–dependent responses. This design enabled a systematic characterization of a microbiota-driven obesity endotype along the oral-gut-liver-brain axis, through which we identified a *Romboutsia_B*-centered risk microbial signature and a relatively stable cluster of metabolically health–associated taxa. Notably, the concordant enrichment of *Romboutsia_B* in both oral and gut samples across ages strengthens the robustness of this signal beyond a single niche and supports its candidacy for noninvasive longitudinal monitoring using oral sampling. These shared salivary and fecal microbial markers may hold value for assessing obesity and its associated risk of impaired brain function.

### Systemic remodeling of the oral-gut-liver-brain axis under long-term high-fat diet

This study found that a high-fat diet induced typical obesity and metabolic-syndrome phenotypes across all age stages, including persistent increases in body weight and Lee’s index, progressive hepatic steatosis and inflammatory infiltration, elevated blood lipids and liver enzymes, and impaired glucose tolerance and insulin sensitivity. These findings are consistent with previous reports of HFD-induced obesity in animal models (Turnbaugh et al., 2006; Tilg et al., 2009; Zhuang et al., 2022; Evans et al., 2024). Furthermore, with increasing age and extended feeding duration, mice on a high-fat diet gradually developed a chronic hypoxic state characterized by declines in SpO_2_, PaO_2_, and SaO_2_, accompanied by elevated serum and cortical Aβ1–42 and LPS levels, as well as spatial learning and memory impairments and exacerbated anxiety- and depressive-like behaviors. These results suggest that excess energy intake resulting from a high-fat diet affects not only the liver and adipose tissues through peripheral lipid accumulation and increased metabolic load but may also impair cerebral perfusion and oxygenation (Grisotto et al., 2021; Zimmerman et al., 2021), activate central neuroinflammatory pathways, and accelerate pathological processes associated with cognitive decline (Boulangé et al., 2016; Lin et al., 2016; Busquets et al., 2017).

At the microbiome level, we observed decreased α-diversity and distinctly shifted beta-diversity clustering patterns in both the oral and gut microbiota of HFD mice across all three age groups, supporting the concept that obesity and its related metabolic abnormalities are accompanied by systemic mucosal microecological remodeling (Bu et al., 2019; Park et al., 2023; Boicean et al., 2025). We found consistent enrichment of inflammation- and higher metabolic-burden–associated taxa such as *Romboutsia* and *Escherichia* in both oral and gut microbiome, whereas metabolic health–associated genera, including *Ruminococcus*, *Bifidobacterium*, and *Akkermansia* were relatively depleted. These findings extend the traditional gut microbiota-obesity-metabolic disease framework toward a more comprehensive oral-gut-liver-brain axis that incorporates oral microbiome and neurocognitive function. This integrative perspective is consistent with recent clinical observations on the oral-gut-cardiometabolic axis (Li et al., 2022; Schamarek et al., 2023) and provides a more systematic microecological basis for understanding obesity-related systemic comorbidities.

### *Romboutsia*: a cross oral-gut marker genus consistently enriched with aging

This study found oral and gut dysbiosis in the HFD compared to the SCD group. Among the differential genera, *Romboutsia* showed consistent enrichment across both oral and gut and at different ages in the long-term high-fat diet mouse model. Correlation analyses indicated that the abundance of *Romboutsia* was closely associated with systemic metabolic alterations, chronic inflammation, and brain functional impairments, including behavioral and cognitive changes. These results suggest that *Romboutsia* may act as a central microbial marker linking the oral-gut microbiome to obesity-related systemic and neurological disturbances.

*Romboutsia*, a strictly anaerobic genus within Peptostreptococcaceae (Gerritsen et al., 2014, Gerritsen et al., 2019), possesses broad metabolic capacities, including carbon utilization, amino acid and vitamin biosynthesis, and bile acid transformation (Gerritsen et al., 2017). Given that the bile acid–microbiota axis is an important mediator of HFD-related metabolic remodeling, these features suggest that Romboutsia may be linked to HFD-related dysmetabolism. Consistent with evidence from large human cohorts and HFD animal models, we found that *Romboutsia_B* correlates with obesity, dyslipidemia, and liver injury, supporting its status as a microbial signature of metabolic dysfunction (Zeng et al., 2019; Li et al., 2021). Nutritional interventions that improve metabolic health often reduce *Romboutsia* while restoring SCFA-producing taxa, suggesting that higher *Romboutsia* is dysbiosis-associated and potentially modifiable in the context of metabolic improvement (Fu et al., 2021; Wang et al., 2021; Hanson et al., 2024; Liang et al., 2024b, Liang et al., 2024a; Luo et al., 2024).

This study also observed that higher *Romboutsia* abundance aligns with lower oxygen saturation. Similar dominance has been reported in HFD models under chronic hypoxia (Ailizire et al., 2023), where *Romboutsia* is linked to reprogramming of energy and amino acid metabolism, reflecting elevated metabolic stress. Taken together, these findings suggest that prolonged HFD, especially when combined with hypoxia, may favor *Romboutsia* expansion, which may be accompanied by higher systemic metabolic load and inflammation, increasing vulnerability to obesity-associated neural impairment. Further metagenomic and interventional studies are needed to validate this hypothesis.

In this study, *Romboutsia_B* was strongly correlated with increased LPS, TNF-α, and Aβ1–42 levels, as well as impaired cognitive behavior, consistent with recent human and animal studies linking *Romboutsia* to gut-brain axis dysregulation. Human studies suggest that reductions in *Romboutsia* may be associated with cognitive benefits: in male patients with type 2 diabetes, metformin significantly reduced *Romboutsia ilealis* abundance, which was positively correlated with memory scores, indicating that suppression of *Romboutsia ilealis* may be a key microbial feature underlying metformin-associated cognitive improvement (Rosell-Díaz and Fernández-Real, 2024; Rosell-Díaz et al., 2024). Additionally, *Romboutsia* is significantly enriched in the gut of Alzheimer’s disease patients and closely associated with hippocampal and amygdala atrophy, representing a potential neuroimaging biomarker (Wanapaisan et al., 2022). Animal studies also show that semaglutide treatment in HFD-induced obese mice improves spatial memory, suppresses hippocampal inflammation, and significantly reverses HFD-induced *Romboutsia* elevation (Feng et al., 2024).

Together with KEGG functional predictions and targeted metabolomics results from this study, these findings suggest a preliminary mechanistic hypothesis: under prolonged high-fat diet conditions, a group of taxa represented by *Romboutsia* increases synchronously in both oral and gut, potentially contributing to microbial metabolic remodeling. This remodeling may be associated with enhanced utilization of fatty acids and ketone bodies, increases amino acid metabolism and DNA damage repair pathway activity, and a potential growth advantage in a high-fat environment. Concurrently, it is associated with a shift in short-chain fatty acid profiles (with increased bcSCFAs and decreased scSCFAs in certain age groups), may be linked to alterations in the bile acid pool, and is associated with higher bacterial component load. Together, these changes may exacerbate hepatic lipid accumulation and inflammation and may contribute to neuroinflammation via the oral-gut-liver-brain axis, linking microbial dysbiosis to metabolic and neural dysfunction under chronic HFD exposure.

### Metabolic imbalance between inflammation-associated and metabolic health–associated microbiota

In this study, *Escherichia* showed similar patterns to *Romboutsia_B*, being enriched in both oral and gut under HFD conditions and broadly correlating with obesity, dyslipidemia, liver enzyme elevation, inflammation, and cognitive impairment, while negatively correlating with blood oxygenation and anti-inflammatory factors. *Escherichia*, a typical Gram-negative Enterobacteriaceae, produces outer membrane LPS, a classical TLR4 agonist that induces pro-inflammatory cytokines such as TNF-α, IL-1β, and IL-6, triggering systemic low-grade inflammation (Alexander and Rietschel, 2001; Beutler and Rietschel, 2003; Cani et al., 2007a). In human studies, *Escherichia-Shigella* is repeatedly reported as enriched in the gut of Alzheimer’s disease and mild cognitive impairment patients, correlating with hippocampal atrophy and cognitive decline (Wanapaisan et al., 2022). We propose that *Escherichia*, via LPS-mediated inflammation, together with *Romboutsia*, may represent a metabolic burden and inflammation amplification microbial axis co-occurrence module correlated with HFD-induced obesity, liver injury, and central nervous system dysfunction.

In contrast to the HFD-enriched taxa, the control mice exhibited significant enrichment of *Ruminococcus_C*, *Bifidobacterium*, *Eubacterium*, *Akkermansia*, and *Muribaculum* in both oral and gut. Their abundances were generally negatively correlated with body weight, blood lipids, liver enzymes, and inflammatory markers, and positively associated with better mood and cognitive performance. *Ruminococcus* and *Eubacterium* are typical SCFA, especially butyrate, producers that support gut barrier integrity and suppress chronic inflammation (Tilg et al., 2009; Ni et al., 2017). *Bifidobacterium* and *Akkermansia* have been repeatedly linked to metabolic health and improved insulin sensitivity in clinical and animal studies (Cani et al., 2007b; Everard et al., 2013; Minami et al., 2018; Depommier et al., 2019), while *Muribaculum* is associated with longevity and favorable lipid metabolism in multiple animal experiments (Smith et al., 2019; Sibai et al., 2020; Du Preez et al., 2021). These findings, consistent with the current results, collectively indicate a metabolic protective role for this group of taxa.

Under HFD conditions, inflammation-associated taxa such as *Romboutsia_B* and *Escherichia* expand, whereas taxa typically linked to metabolic health, including *Ruminococcus_C*, *Bifidobacterium*, *Eubacterium*, *Akkermansia*, and *Muribaculum* are relatively depleted. This pattern is consistent with a shift of the oral and gut microbiota from an SCFA-producing, barrier-maintaining state toward an inflammation-associated, lipid- and bile acid-burdened state. Within this framework, *Romboutsia_B* and its co-occurring taxa should not be viewed as singular pathogens but as representative members of a dysbiosis-associated microbial community and functional network.

Accordingly, future microbiome-targeted interventions could go beyond the linear strategy of merely suppressing pro-inflammatory taxa and supplementing SCFA-producing beneficial taxa. Instead, approaches such as dietary modification, functional foods, probiotics, and synbiotics may help enhance SCFA production and barrier-protective modules at the community level while attenuating the *Romboutsia_B*-associated dysbiosis module, potentially reducing obesity-related metabolic burden and supporting the prevention or delay of associated cognitive and neurological dysfunction.

The study has several strengths. First, the comprehensive, multi-age design spans the adult lifespan, allowing the assessment of aging interactions with HFD on metabolism, microbiota, and systemic impairment. Second, by integrating multi-organ phenotyping, oral and gut microbiota profiling, metabolomics, and multidimensional behavioral testing, it provides a systemic view linking microbiota changes to metabolic and brain function. Third, the consistent cross-oral-gut associations, particularly of *Romboutsia_B*, enhance the translational relevance of the findings. However, this study also has some limitations. First, this study relied on 16S rRNA sequencing, which resolves taxonomy mainly at the genus level, limiting the ability to distinguish functionally distinct strains. Second, functional predictions based on KEGG are inferential, and we did not comprehensively measure central or peripheral transcriptomes/proteomes or directly quantify bile acid profiles and related host pathways. Third, the study used only male C57BL/6J mice with a limited sample size, leaving uncertainty about generalizability across sexes, strains, and genetic backgrounds. Fourth, as an observational study, the causal direction of the microbiota-metabolism/inflammation-behavior network cannot be established, and confounding upstream factors cannot be excluded; experimental manipulations are needed to clarify the role of *Romboutsia_B*.

## Conclusions

Long-term high-fat diet induces systemic remodeling of the oral-gut-liver-brain axis in mice. *Romboutsia_B* was consistently enriched in both oral and gut microbiota and correlated with obesity, liver injury, hypoxia, and cognitive impairments, while metabolic health–associated genera were depleted. These shifts were associated with altered lipid and SCFA metabolism and increased inflammatory load, suggesting that *Romboutsia_B* may serve as a noninvasive microbial biomarker associated with obesity-related liver–brain comorbidities and warrants validation in longitudinal and interventional studies.

## Data Availability

The datasets presented in this study can be found in online repositories. The names of the repository/repositories and accession number(s) can be found in the article/**Supplementary Material**.
