# Supplementary material for: Role of oral-gut microbiota dysbiosis in regulating systemic impairment during age-related obesity: an animal study
Source: Front Cell Infect Microbiol. 2026 Feb 26;16:1781222. doi: 10.3389/fcimb.2026.1781222 (PMC12979499; doi:10.3389/fcimb.2026.1781222)
Supplement: Supplementary file 1 [file DataSheet1.docx]

**SUPPLEMENTARY MATERIAL TO**

**Role of oral-gut microbiota dysbiosis in regulating systemic impairment**

**during age-related obesity: an animal study**

Yixue Tian^1,2,3,#^, Min Yu^1,2,3,#^, Jingxuan Bai^1,2,4^, Yuke Chen^1,2,3^, Xin Cong^5,*^, Xuemei Gao^1,2,3,*^

^1^ Department of Orthodontics, Peking University School and Hospital of Stomatology, No. 22 Zhongguancun South Avenue, Haidian District, Beijing, 100081, P.R. China

^2^ Center for Oral Therapy of Sleep Apnea, Peking University Hospital of Stomatology, No. 22 Zhongguancun South Avenue, Haidian District, Beijing, 100081, P.R. China

^3^ National Center for Stomatology, No. 22 Zhongguancun South Avenue, Haidian District, Beijing, 100081, P.R. China

^4^ Dental Medical Center, China-Japan Friendship Hospital, No. 2 Yinghuayuan East Street, Chaoyang District, Beijing, 100029, P.R. China

^5^ Department of Physiology and Pathophysiology, Peking University Health Science Center, Haidian District, Beijing, 100191, P.R. China

^#^ The first two authors contributed equally to this work.

Corresponding authors: Xin Cong and Xuemei Gao contributed equally to this work.

Xin Cong, Research Fellow, Department of Physiology and Pathophysiology, Peking University Health Science Center, No. 38 Xueyuan Road, Haidian District, Beijing, 100191, P.R. China (E-mail: [congxin@bjmu.edu.cn](mailto:congxin@bjmu.edu.cn), Telephone: 86-010-82802403)

Xuemei Gao, Professor, Department of Orthodontics, Center for Oral Therapy of Sleep Apnea, Peking University School and Hospital of Stomatology & National Center of Stomatology & National Clinical Research Center for Oral Diseases & National Engineering Laboratory for Digital and Material Technology of Stomatology & Beijing Key Laboratory for Digital Stomatology & Research Center of Engineering and Technology for Computerized Dentistry Ministry of Health & NMPA Key Laboratory for Dental Materials, No. 22 Zhongguancun South Avenue, Haidian District, Beijing, 100081, P.R. China (E-mail: [xmgao@263.net](mailto:xmgao@263.net), Telephone: 86-010-82195350, Fax: 86-010-82195350; ORCID: 0000-0001-5690-9385)

**Table of contents**

[**Supplementary 1. Details of the standard chow diet and the high-fat diet** 3](#_Toc221024169)

[**Supplementary 2. Methods of Behavior Tests** 3](#_Toc221024170)

[**Supplementary 3. Arterial Blood Gas Analysis of the Abdominal Aorta in Middle and Old Age Group Mice** 6](#_Toc221024171)

[**Supplementary 4. Serum and Cortex IL-6 and IL-10 Levels** 8](#_Toc221024172)

[**Supplementary 5. KEGG pathway functional prediction of the oral and gut microbiomes** 9](#_Toc221024173)

### **Supplementary 1. Details of the standard chow diet and the high-fat diet**

During the experimental period, mice were fed either a standard chow diet (SCD) or a high-fat diet (HFD). The SCD (XTI01WC-010; Jiangsu Xietong Pharmaceutical Bioengineering Co., Ltd., China) provided an energy distribution of 11.1% kcal from fat, 21.5% kcal from protein, and 67.4% kcal from carbohydrates. According to the manufacturer’s information, soybean oil was the primary lipid source, and no cholesterol supplementation was indicated.

The HFD was D12492 (Research Diets, Inc., New Brunswick, NJ, USA), providing 60% kcal from fat, 20% kcal from protein, and 20% kcal from carbohydrates, with an energy density of approximately 5.21 kcal/g. The primary fat sources were lard (major) and soybean oil (minor), consistent with the publicly available formulation.

No cholesterol was added as a separate ingredient; the measured cholesterol content reported for D12492 is attributable mainly to animal-derived ingredients (primarily lard, with minor contribution from casein) and is typically listed at ~280 mg/kg in manufacturer product data.

Both diets were irradiated before use. Diets were stored at −20°C and protected from moisture and light. After opening, each diet bag was used within 1 month to minimize nutrient oxidation and batch variability. Fresh diet and autoclaved drinking water were provided ad libitum throughout the study.

### **Supplementary 2. Methods of Behavior Tests**

All behavioral experiments were conducted in a quiet room between 8:00 AM and 17:00 PM. Mice were transferred to the testing room 1 hour before each experiment for habituation. Except for the Morris water maze (performed with curtains drawn) and the forced swim test (performed with lights on), all behavioral tests were carried out with the room lights turned off and illuminated solely by an infrared supplemental light source (GY-IR110) for night-vision recording. An industrial camera (JHSM130Bs-R) was used to record all tests, and mouse trajectories were analyzed using SMART software (v3.0, Panlab). After each trial, all arenas and apparatuses were thoroughly cleaned with 70% ethanol to remove olfactory cues. All behavioral tests were performed and analyzed by the same researcher. Different behavioral tests were scheduled 24 to 72 hours apart to allow sufficient rest and recovery.

Behavioral testing and scoring were performed by an investigator blinded to group allocation (animals were coded prior to testing), and the same blinded investigator conducted data extraction/analysis to ensure procedural consistency across assays. The behavioral battery followed a fixed sequence, as indicated by arrows in the “behavior test” module of the study design (Figure 1): OFT → NOR → YM → TCST → EPM → FST → MWM. To minimize carryover effects related to stress, fatigue, and learning, predefined inter-assay intervals were implemented: low-stress assays (OFT, NOR, YM) were separated by 24 h, whereas higher-arousal/learning-intensive assays (TCST, EPM, FST, and the start of MWM) were separated by 48 h.

**Open Field Test (OFT)**

Mice were allowed to freely explore the open field for 8 minutes. The time spent and the distance traveled in the central area, the number of entries into the center, and the total distance traveled in the arena were quantified to assess exploratory behavior, locomotor activity, and anxiety-like behavior in a novel environment.

**Novel Object Recognition Test (NOR)**

The NOR test evaluates short-term memory and cognitive function based on rodents’ natural preference for novelty. On day 1, mice freely explored an empty open arena for 8 minutes for habituation. On Day 2, two identical cubes were placed in opposite corners of the arena. Mice explored for 5 minutes for training, and the time spent exploring each object was recorded. Two hours later, one cube was replaced with a cone for testing. Mice explored for 5 minutes, and the time spent exploring the novel (TN) and familiar objects (TF) was recorded. The discrimination index (DI) was calculated as DI = [(TN − TF) / (TN + TF)] × 100%.

**Y-Maze (YM)**

The Y-maze consists of three opaque arms arranged at 120° angles. Mice were placed at the center of the maze and allowed to explore freely for 8 minutes. A spontaneous alternation was defined as consecutive entries into three different arms. The percentage of spontaneous alternation was used to assess working memory.

**Three-Chamber Social Test (TCST)**

A stranger mouse (S1) was placed in a metal cage in one side chamber, while the cage on the opposite side remained empty (E). The test mouse was placed in the central chamber and allowed to explore for 10 minutes. Two hours later, a second unfamiliar mouse (S2) was placed into the previously empty cage. The test mouse explored for 10 minutes. Time spent in contact with E, S1, or S2 was recorded. Sociability index = [(S1 - E) / (S1 + E)] × 100%, and social novelty preference index = [(S2 - S1) / (S2 + S1)] × 100%. (Figure A)

**Elevated Plus Maze (EPM)**

The maze consists of two open arms and two closed arms arranged in a plus shape, elevated 50cm above the floor. Mice were placed in the center facing an open arm and allowed to explore for 5 minutes. The number of entries and time spent in the open and closed arms were recorded to evaluate anxiety-like behavior.

**Forced Swim Test (FST)**

Mice were placed in a cylindrical container (height 25cm, diameter 12cm) filled with 16cm of water. The test lasted 6 minutes, and immobility time during the final 4 minutes was quantified. Immobility was defined as floating passively with minimal limb movement sufficient only to keep the head above water.

**Morris Water Maze (MWM)**

The maze consisted of a 120cm diameter circular pool filled with water made opaque using nontoxic titanium dioxide (10022728). On Day 1, the platform was placed protruding 1cm above the water surface. Each mouse was released from points D and F facing the wall. If it failed to find the platform within 60 seconds, it was guided to it and allowed to remain for 15 seconds.

On Days 2 to 5, the platform was submerged 1cm below the surface. Each mouse underwent training trials starting from points D, F, C, and G with 20-minute intervals. On Day 6, the platform was removed. Mice were released from point E and allowed to swim for 60 seconds.

Trajectory recordings and SMART 3.0 analysis were used to assess latency to reach the hidden platform during acquisition, as well as swim path, number of crossings over the former platform location, and time spent in the target quadrant during the probe trial, which were analyzed as indices reflecting hippocampus-dependent spatial learning and memory. (Figure B)


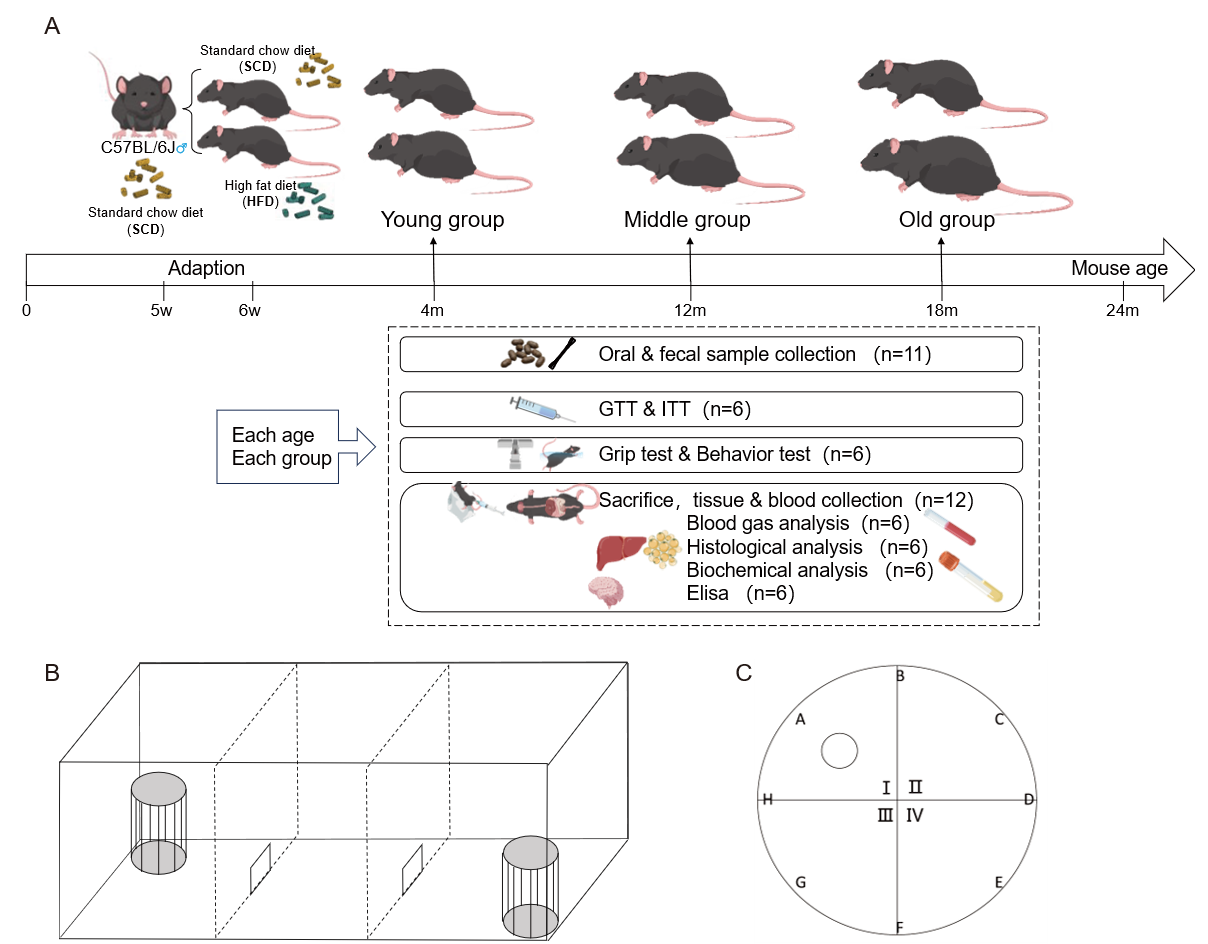


**A**

**B**

### **Supplementary 3. Arterial Blood Gas Analysis of the Abdominal Aorta in Middle and Old Age Group Mice**

| Parameters | middle SCD  (n=6) | middle HFD  (n=6) | *P* | old SCD  (n=7) | old HFD  (n=6) | *P* |
| --- | --- | --- | --- | --- | --- | --- |
| pH | 7.250±0.038 | 7.275±0.054 | 0.387 | 7.317±0.075 | 7.303±0.069 | 0.756 |
| PaO_2_ (mmHg) | 188.833±26.746 | 148.500±56.156 | 0.143 | 137.000±15.232 | 94.000±40.694 | 0.036* |
| PaCO_2_ (mmHg) | 26.850±8.700 | 29.983±5.971 | 0.484 | 27.000±5.096 | 34.650±6.508 | 0.047* |
| Hematocrit (%) | 39.667±2.160 | 38.500±4.930 | 0.607 | 37.500 (31.250, 40.000) | 34.500 (30.750, 35.200) | 0.124 |
| Electrolytes / metabolites (mmol/L) | | | | | | |
| Na^+^ | 154.500±1.049 | 153.500±4.087 | 0.574 | 156.333±3.077 | 158.500±4.037 | 0.320 |
| K^+^ | 4.092±0.290 | 4.022±0.192 | 0.658 | 3.135 (2.642, 3.395) | 3.655 (2.810, 4.418) | 0.200 |
| Ca^2+^ | 1.320 (1.283, 1.348) | 1.220 (0.893, 1.270) | 0.019* | 0.888 ± 0.260 | 0.762 ± 0.444 | 0.563 |
| Cl^-^ | 124.500 (121.750, 125.250) | 123.000 (119.500, 128.500) | 0.872 | 126.833±5.456 | 127.167±5.707 | 0.920 |
| Lactate | 1.467±0.216 | 1.633±0.638 | 0.558 | 1.267±0.550 | 1.533±0.769 | 0.505 |
| Total hemoglobin  (tHb, g/dL) | 12.757±0.680 | 12.550±1.694 | 0.771 | 12.100 (10.025, 12.900) | 11.100 (9.900, 11.375) | 0.107 |
| HCO^3-^ (act, mmol/L) | 14.133±1.214 | 14.083±1.677 | 0.954 | 11.983±4.450 | 12.267±4.990 | 0.919 |
| HCO^3-^ (std, mmol/L) | 15.317±1.046 | 14.983±1.389 | 0.649 | 16.000 (12.375, 17.275) | 14.000 (11.955, 16.925) | 0.423 |
| Base Excess (mmol/L) | -11.883±1.472 | -12.333±1.989 | 0.665 | -10.900 (-16.000, -9.050) | -13.550 (-16.550, -9.050) | 0.047 |
| Total carbon dioxide (TCO₂, mmol/L) | | | | | | |
| Whole blood  ctCO2(B) | 13.083±1.148 | 13.167±1.579 | 0.919 | 12.100 (8.825, 13.475) | 9.250 (8.400, 15.875) | 0.631 |
| Plasma  ctCO2(P) | 15.133±1.274 | 15.150±1.832 | 0.986 | 12.683±4.723 | 13.100±5.348 | 0.889 |
| Standardized calcium ion concentration  Ca^2+^(7.40), (mmol/L) | 1.208±0.034 | 1.144±0.035 | 0.013* | 0.865 (0.640, 1.063) | 0.745 (0.315, 1.105) | 0.631 |
| Anion Gap (mmol/L) | 16.600 (15.575, 18.875) | 17.100 (12.920, 20.100) | 0.749 | 17.467±2.951 | 18.950±3.292 | 0.430 |
| Oxygen saturation  sO_2_ (%) | 99.183±0.450 | 97.850±2.096 | 0.159 | 98.833 ± 0.501 | 94.500 ± 4.355 | 0.036* |
| Arterial oxygen content  ctO2 (vol%) | 18.217±0.972 | 17.283±3.001 | 0.485 | 16.617±1.567 | 13.800±2.044 | 0.023* |

**p*<0.05

### **Supplementary 4. Serum and Cortex IL-6 and IL-10 Levels**


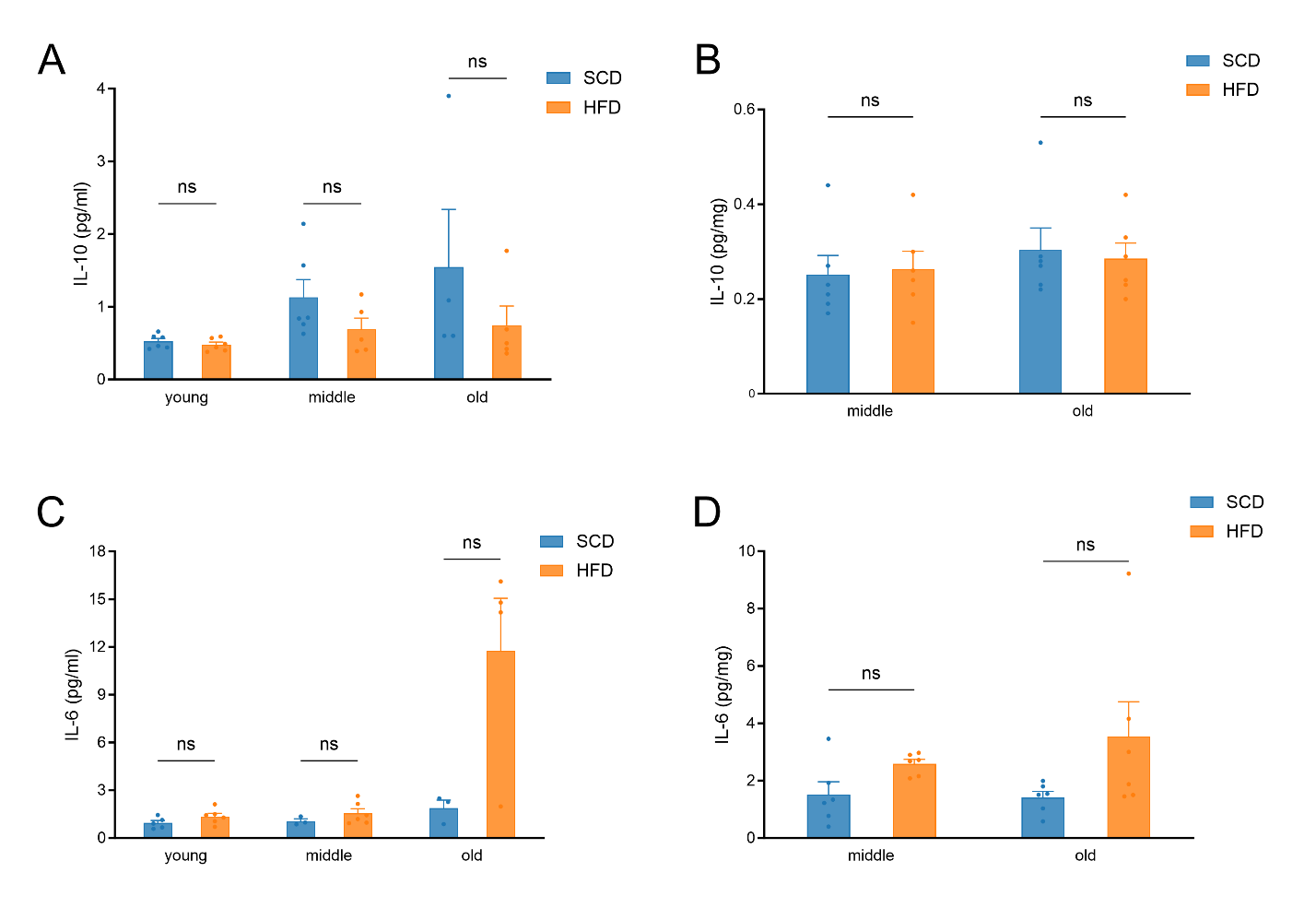


A. Serum interleukin-10 (IL-10) level of mice in young, middle, and old age groups;

B. Cortex IL-10 level of mice in the middle and old age groups;

C. Serum IL-6 level of mice in young, middle, and old age groups;

D. Cortex IL-6 level of mice in the middle and old age groups.

SCD: standard-chow diet; HFD: high-fat diet; ns: non-significant.

###
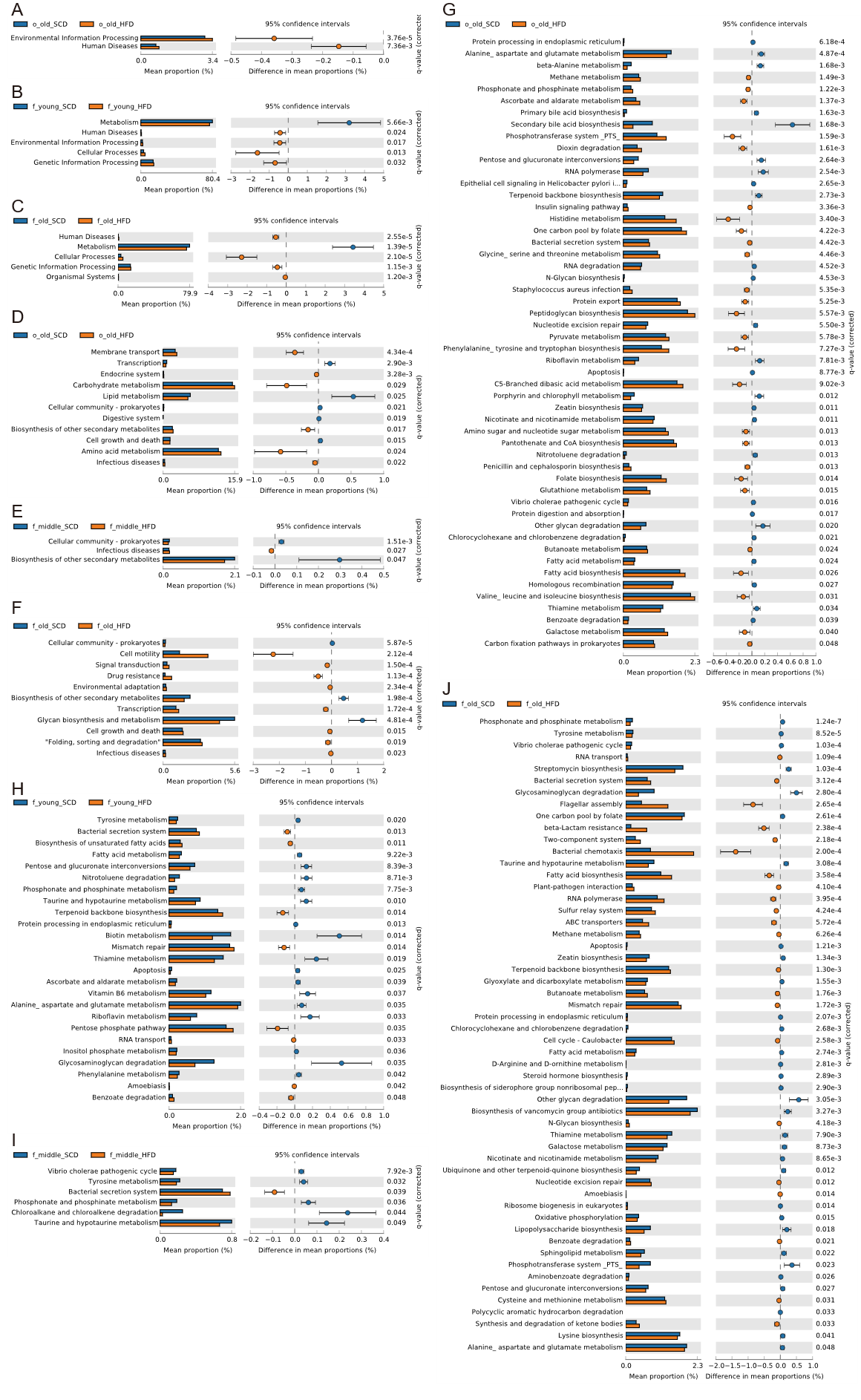
**Supplementary 5. KEGG pathway functional prediction of the oral and gut microbiomes**

A to C. L1-level KEGG pathway functional metabolic predictions
(A) The oral microbiome in the old group; (B) The gut microbiome in the young group; (C) The gut microbiome in the old group.

D to F. L2-level KEGG pathway functional metabolic predictions
(D) The oral microbiome in the old group; (E) The gut microbiome in the middle group; (F) The gut microbiome in the old group.

G to J. Pathway-level KEGG pathway functional metabolic predictions
(G) The oral microbiome in the old group; (H) The gut microbiome in the young group; (I) The gut microbiome in the middle group; (J) The gut microbiome in the old group.

SCD: standard chow diet; HFD: high-fat diet.
